# Case presentation: a severe case of cobalamin c deficiency presenting with nephrotic syndrome, malignant hypertension and hemolytic anemia

**DOI:** 10.1186/s12882-024-03656-1

**Published:** 2024-07-08

**Authors:** Halil Tuna Akar, Harun Yıldız, Zeynelabidin Öztürk, Deniz Karakaya, Abdullah Sezer, Asburçe Olgaç

**Affiliations:** 1 Department of Pediatric Metabolism, Ankara Etlik City Hospital Health Complex Children’s Hospital, Ankara, Turkey; 2 Department of Pediatric Intensive Care Medicine, Ankara Etlik City Hospital, Ankara, Turkey; 3 Department of Pediatric Nephrology, Ankara Etlik City Hospital, Ankara, Turkey; 4 Deparment of Medical Genetics, Ankara Etlik City Hospital, Ankara, Turkey

**Keywords:** Inborn errors of metabolism, Cobalamin C deficiency, *MMACHC*, Nephritic syndrome, Hemolytic anemia

## Abstract

**Background:**

The etiology of nephrotic syndrome can vary, with underlying metabolic diseases being a potential factor. Cobalamin C (cblC) defect is an autosomal recessive inborn error of metabolism caused by mutations in the *MMACHC* gene, resulting in impaired vitamin B12 processing. While cblC defect typically manifests with hematological and neurological symptoms, renal involvement is increasingly recognized but remains rare.

**Case Presentation:**

We describe a 7-month-old male patient presenting with fatigue and edema. His initial laboratory findings showed anemia, thrombocytopenia, hypoalbuminemia and proteinuria. Further examinations reveals hemolysis in peripheral blood smear. During his follow up respiratory distress due to pleural effusion in the right hemithorax was noticed. And fluid leakage to the third spaces supported a diagnosis of nephrotic syndrome. The patient’s condition deteriorated, leading to intensive care admission due to, hypertensive crisis, and respiratory distress. High total plasma homocysteine and low methionine levels raised suspicion of cobalamin metabolism disorders. Genetic testing confirmed biallelic *MMACHC* gene mutations, establishing the diagnosis of cblC defect. Treatment with hydroxycobalamin, folic acid, and betaine led to remarkable clinical improvement.

**Discussion/Conclusion:**

This case underscores the significance of recognizing metabolic disorders like cblC defect in atypical presentations of nephrotic syndrome. Early diagnosis and comprehensive management are vital to prevent irreversible renal damage. While cblC defects are more commonly associated with atypical hemolytic uremic syndrome, this case highlights the importance of considering cobalamin defects in the differential diagnosis of nephrotic syndrome, especially when associated with accompanying findings such as hemolysis. Our case, which has one of the highest homocysteine levels reported in the literature, emphasizes this situation again.

## Introduction

Nephrotic syndrome is a complex clinical entity characterized by proteinuria, hypoalbuminemia, edema, and hyperlipidemia [1]. While nephrotic syndrome is commonly associated with primary renal disorders such as minimal change disease, focal segmental glomerulosclerosis, and membranous nephropathy, and rare etiological causes should be considered.

Nephrotic syndrome can affect people of all ages, but boys are more affected [2]. In nephrotic syndrome, protecting the kidneys, preventing complications, treatment and disease management are very important [1]. Treatment includes controlling complications and treating the medical condition causing nephrotic syndrome. Disease-related complications include edema, infections, thromboembolism, hypovolemic crisis, anemia, and acute renal failure. Although idiopathic nephrotic syndrome has an important place in the pediatric age group, if other accompanying system findings are present, it is necessary to investigate other etiological causes [3]. One of these rare etiological causes in childhood is Cobalamin-C defect, which is a B12 metabolism disorder. Kidney involvement in cobalamin-C deficiency results in glomerular and tubular injury. Most of the patients present with clinical findings such as renal thrombotic microangiopathy (TMA) associated with acute renal failure and typical lesions of renal thrombotic microangiopathy on kidney biopsy are different from idiopathic nephrotic syndrome clinic. At the same time, the management of the disease and response to treatment also vary.

In this case report, we present a rare and intriguing clinical scenario of a patient with nephrotic syndrome, malign hypertension hemolytic anemia attributed to Cobalamin C (cblC) defect. it is essential for clinicians to be aware of its diverse etiologies, including rare metabolic disorders like cblC defect [4].

## Case

A 7-month-old male patient was admitted to our center because of the need for intensive care due to pleural effusion and respiratory distress from a different center. In his history, it was learned that the patient had been admitted to the pediatrics clinic with complaints of fatigue for 1–2 months and was hospitalized for investigation. Pancytopenia was detected at the first admission. Serum creatinine was normal. Chest radiography was evaluated as normal. Renal USG showed renal parenchymal hyperecogenity favoring renal parenchymal disease. The patient was evaluated for the etiology of non-immune hemolytic anemia during follow-up. Peripheral blood smear showed no blasts. Normochromia and marked anisocytosis were observed in the morphology. Diffuse fragmented erythrocytes and rare spherocytes were observed. In terms of malignancy, contrast-enhanced computed tomography of the thorax and abdomen showed no mass appearance.

In the patient’s bone marrow aspiration, heterogeneous normocellular bone marrow appearance was detected. Bone marrow elements were observed in all three cell lines. No foreign cell infiltration was observed. While he was still hospitalized, a full urinalysis revealed 15 erythrocytes per high magnification field and + + proteinuria. In the patient’s follow-up physical examination, ascites was not balloted, but peripheral edema (pretibial edema) was present. In biochemical examinations, the protein-creatinine ratio in spot urine was found to be 4373.08 mg/g creatinine. Total protein in the blood was found to be low as 3.74 g/dl and albumin as 1.83 g/dl. On admission, his serum triglyceride value was found to be within normal limits as 78 mg/dl. With the clinical and laboratory findings available, the patient was primarily evaluated for nephrotic syndrome. The patient was taken to intensive care due to increased parenchymal echogenicity and nephromegaly seen on sonography and hypertensive crisis. The patient’s fundus examination revealed findings consistent with stage 1–2 retinopathy. No pathological findings were detected on cranial mangetic resonance imaging (MRI). In the thorax ultrasound performed due to desaturation during follow-up, a pleural effusion of 45 mm on the right and 10 mm on the left was detected. A thorax tube was inserted. The patient was referred to our hospital, which is an advanced center, with the plan to determine the etiology of renal pathology and perform a renal biopsy.

During the physical examination of the patient upon arrival at our center, it was observed that he tended to sleep and his breathing sounds were decreased at the base of the right lung. The patient had respiratory distress and was observed to have intercostal and subcostal retractions. The spleen was palpated one cm below the rib in the midclavicular line. The patient’s peripheral blood smear showed normochrome anisocytic erythrocytes. Again, schistocytes, bite cells and mild polychromasia were seen in the smear, but no atypical cells were seen. The hemoglobin value was seen as 5.7 g/dl in the blood count. Erythrocyte transfusion was performed.

In the following days, intubation and positive pressure ventilation were required due to respiratory distress due to pleural effusion in the right hemithorax. The patient required intravenous esmolol infusion due to accompanying malignant hypertension. The patient with hematuria, proteinuria, resistant hypertension and hemolytic anemia was evaluated in terms of possible inborn errors of metabolism (IEM). The patient’s parents were unrelated, and there was no notable medical history within the family. Basic metabolic scans were requested.

The patient’s total plasma homocysteine was found to be 1700.5 micromol/L (Normal < 10). Simultaneously, the blood vitamin B12 level was determined to be 217 ng/L. From a clinical perspective, it was thought that it might be compatible with clinically atypical nephrotic syndrome.

In the differential diagnosis, cobalamin metabolism disorders were considered prominently.In the acylcarnitine profile, C3 propionyl carnitine was detected as 9.16 mmol/L (Normal < 1.5). In plasma amino acid analysis, methionine was found to be low at 2.85 micromol/L. In urine organic acid analysis, methylmalonic acid (MMA) excretion was found to be high as 530 mmol/mol creatinine (< 11).

**Fig. 1:**
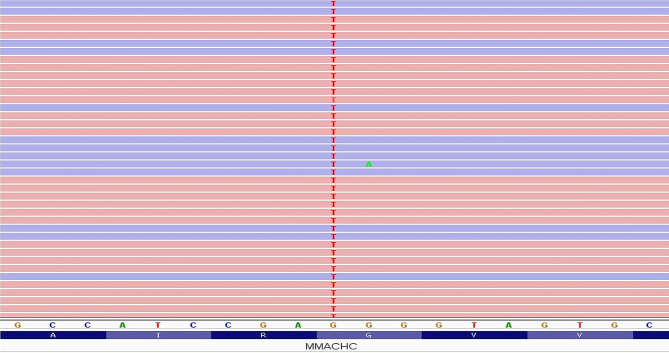
Sequence view

Based on the patient’s present clinical findings, along with markedly elevated homocysteine and C3 propionyl carnitine levels, and decreased methionine levels, a diagnosis of Cobalamin C deficiency was established both clinically and biochemically.The patient was started on 1 mg/day IM hydroxycobalamin and folic acid. Betaine was given orally at a dose of 100 mg/kg/day in three equal doses. Treatment doses were doubled because the total homocysteine level increased to extreme values of 232,000 micromol/L. 2 mg/day hydroxycobalamine was continued at 0.22 mg/kg/day dosage. A next-generation sequencing panel analysis was conducted with a focus on the etiology associated with methylmalonic aciduria, involving the genes *ABCD4*, *HCFC1*, *LMBRD1*, *MMACHC*, and *MMADHC*. In the analysis, the c.484G > T:p.(Gly162Trp) variant was identified in the homozygous state in the *MMACHC* gene (NM_015506.3) (Fig. 1). The patient’s parents examined within the scope of the segregation study were found to be heterozygous carriers. This variant, registered as rs1178984269 in the dbSNP database, lacked entries in the gnomAD and ClinVar databases. It was first reported in the literature in a homozygous state in a patient with multisystemic disease presenting with failure to thrive, developmental delay, hypotonia, visual impairment, hematologic manifestations, and pulmonary hypertension in 2014 (PMID: 24853097). Another study reported the same variant in a homozygous state in a patient with chronic thrombotic microangiopathy-induced renal damage and acute hemolytic lesions (PMID: 29068997). The affected p.Gly162 amino acid residue in the patient was located in a hotspot region where missense pathogenic variants were clustered. In silico tools (Alphamissense, Revel, and MetaRNN) predicted the c.484G > T:p.(Gly162Trp) variant as disease-causing. Based on all this information, this variant was assessed according to the recommendation of the ACMG 2015 variant classification guidelines (PMID: 25,741,868) and classified as pathogenic, consistent with the diagnosis of methylmalonic aciduria and homocystinuria, cblC type.

The patient, demonstrating amelioration of nephrotic syndrome and pancytopenia, alongside regression of respiratory distress, has been discharged and is presently under close medical surveillance. The patient’s blood pressure remained within normal range while receiving treatment consisting of 5 mg amlodipine daily, 5 mg propranolol twice daily, and 1 mg doxazosin twice daily. The patient’s hydroxycobalamin maintenance treatment was continued at 1 mg intramuscularly every other day. At the last follow-up visit, homocysteine decreased to values as low as 4.6 micromol/L, but low methionine persisted.

## Discussion

Cobalamin C defect is an autosomal recessive inborn error of metabolism caused by mutations in the MMACHC gene, leading to impaired intracellular processing of vitamin B12 (cobalamin). The consequence of this deficiency extends beyond its well-known effects on hematopoiesis and neurological function; it can also affect various organ systems, including the kidneys. Kidney involvement in cblC defect is not common but has been increasingly recognized in recent years [5]. Renal manifestation as the primary symptom of cblC deficiency is infrequent, and the routine measurement of both plasma homocysteine and urine methylmalonic acid is not common practice. Consequently, cblC-related renal disorders are often misdiagnosed. Therefore, it is proposed that screening for cblC deficiency be considered in patients presenting with either ambiguous intravascular hemolysis, hematuria, and proteinuria, or renal thrombotic microangiopathy (TMA) [5, 6].

Cobalamin-C (CblC) deficiency is an autosomal recessive disease, and the most common B12 metabolism disorder. CblC is a cofactor of methylmalonyl-CoA mutase and methionine synthase. Methylmalonyl-CoA mutase converts L-methylmalonyl-CoA to succinylCoA in mitochondria. Methionine synthase converts homocysteine into methionine in the cytosol [7, 8]. Defects in the synthesis of these two enzymes lead to hyperhomocysteinemia and methylmalonic aciduria. Hyperhomocysteinemia is responsible for vascular findings [9, 10].

It often manifests in the neonatal period with vomiting, decreased sucking, lethargy, hypotonia, thrombocytopenia, renal failure and hemolytic anemia. In addition, respiratory failure, hepatic failure and gastrointestinal bleeding may occur. Although the disease often presents in the neonatal period, older cases have also been reported [11].

While serious neurological, ocular, hematological, renal, gastrointestinal and cardiac involvements are observed in early-onset patients; symptoms are mild, acute/slowly progressive neurological and behavioral problems may occur in late-onset patients,. Hydroxycobalamin, folinic acid, L-carnitine and betaine are used in the treatment [12].

The pathophysiology of renal involvement in cblC defect is complex and not fully elucidated. Proposed mechanisms include the deposition of abnormal metabolites within glomerular and tubular structures, endothelial dysfunction, and immune-mediated processes [6]. These mechanisms can collectively lead to glomerular and tubular injury, ultimately resulting in nephrotic syndrome, as seen in our patient.

Our patient presented with the classical features of nephrotic syndrome, including severe proteinuria, hypoalbuminemia and edema. Laboratory investigations revealed marked proteinuria, hypoalbuminemia, and hypercholesterolemia, which are hallmarks of nephrotic syndrome. The diagnosis of cblC defect was confirmed through genetic testing, which identified biallelic mutations in the *MMACHC* gene. Additionally, very elevated levels of plasma homocysteine and methylmalonic acid excretion in our patient’s urine were consistent with the biochemical abnormalities associated with this disorder.

The management of cblC defect with nephrotic syndrome is multifaceted and challenging. Treatment strategies aim to correct the metabolic derangements associated with vitamin B12 deficiency while addressing the renal manifestations. Lifelong vitamin B12 supplementation is the cornerstone of therapy, typically administered intramuscularly or subcutaneously. In addition to vitamin B12, other nutritional deficiencies should be assessed and corrected. Moreover, kidney-specific interventions, such as angiotensin-converting enzyme inhibitors or angiotensin II receptor blockers, may be necessary to manage proteinuria and hypertension [13]. The long-term prognosis of nephrotic syndrome in cblC defect is variable and often depends on the extent of renal involvement and the promptness of treatment initiation. Early diagnosis and appropriate management are crucial to prevent or mitigate irreversible kidney damage and improve overall clinical outcomes.

It has been reported that cobalamin defects are mostly involved in the pathogenesis of atypical HUS in the renal system. However, in our case, the presentation with atypical nephrotic syndrome clinic draws attention as atypical. However, the patient’s significant anemia should also make cobalamin defects considered in the differential diagnosis. Total plasma homocysteine measurement can be considered as an initial screening test in patients with other system involvement as well as nephrotic syndrome [14].

Malignant hypertension was observed in our patient during his clinical course, and malignant hypertension in cobalamin c mutation has been previously reported in the literature [15].

**Fig. 2:**
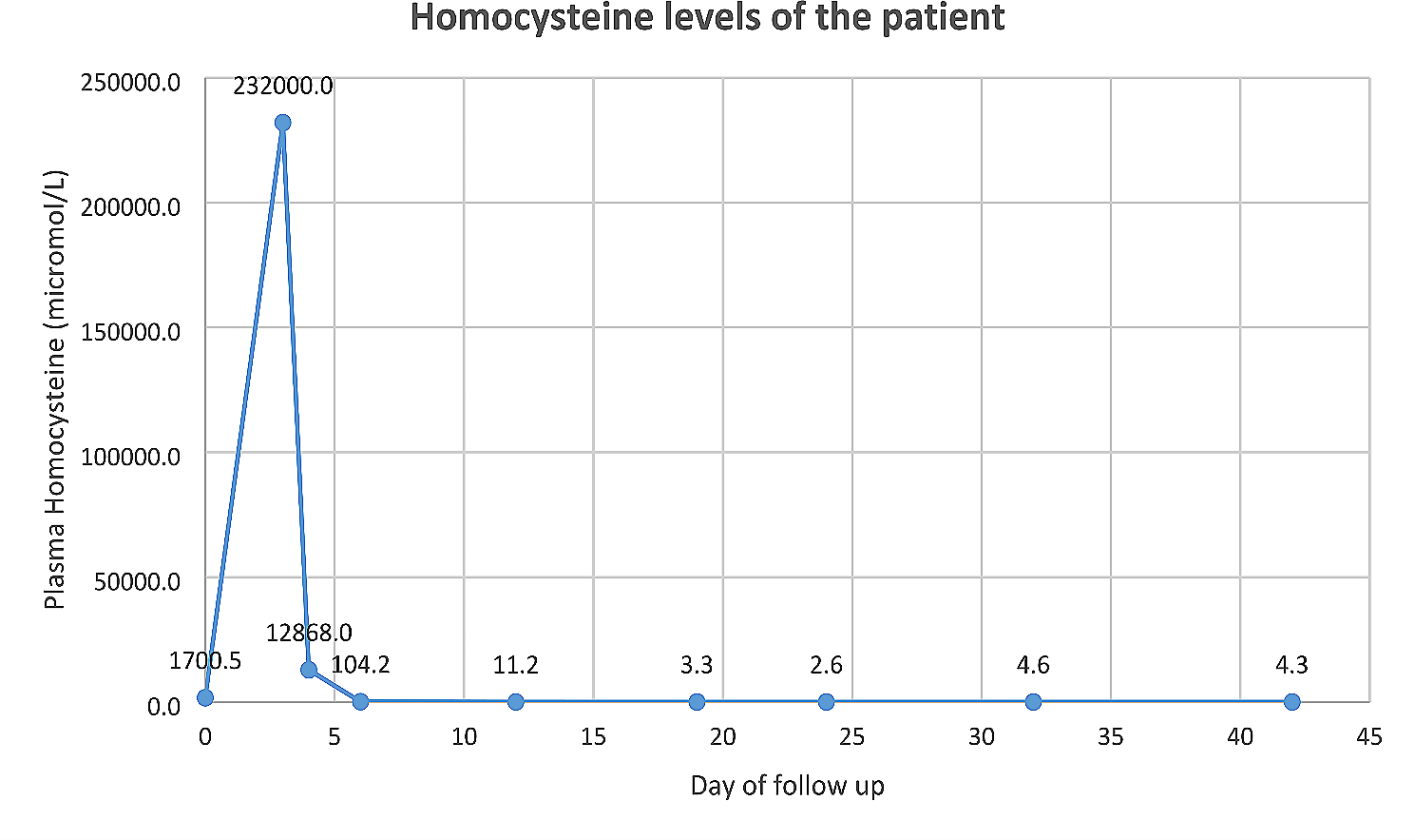
Homocysteine levels of the patient

The first homocysteine level detected in our patient started with 1700 mmol/L, rose to unimaginably high values of 232,000, and then decreased to 66 mmol/L within 3 days with effective treatment, showing how effective betaine and hydroxycobalamin supplements are. (Fig. 2) [16].

In conclusion, this case underscores the importance of considering metabolic disorders such as cblC defect in the differential diagnosis of nephrotic syndrome, particularly in cases with atypical or severe presentations. Timely recognition and management of these rare conditions are essential to prevent complications and improve the quality of life for affected individuals. Further research is needed to better understand the mechanisms underlying renal involvement in cblC defect and to optimize treatment strategies for affected patients.

## Data Availability

All data used for the case report are included in this published article. The genetic study was carried out in our center. Raw data in our in-house database can be shared-on demand if the corresponding author is contacted. The datasets generated and/or analyzed during the current study are available in the ClinVar repository, (https://www.ncbi.nlm.nih.gov/clinvar/, submission number SUB13957605) ”.
